# Identification and functional analysis of cation-efflux transporter 1 from *Brassica juncea* L.

**DOI:** 10.1186/s12870-022-03569-x

**Published:** 2022-04-06

**Authors:** Lu Han, Xiaohua Wu, Xinyu Zhang, Kailin Hou, Hongshan Zhang, Chenjia Shen

**Affiliations:** 1 College of Life and Environmental Sciences, Hangzhou Normal University, Hangzhou, 310036 China; 2 Zhejiang Key Laboratory of Organ Development and Regeneration, College of Life and Environmental Sciences, Hangzhou Normal University, Hangzhou, 310036 Zhejiang China; 3 Zhejiang Provincial Key Laboratory for Genetic Improvement and Quality Control of Medicinal Plants, Hangzhou Normal University, Hangzhou, 310036 China

**Keywords:** *Brassica juncea*, Cation-efflux transporter, Heavy metal ion, Heavy metal tolerance, Heterologous expression

## Abstract

**Background:**

*Brassica juncea* behaves as a moderate-level accumulator of various heavy metal ions and is frequently used for remediation. To investigate the roles of metal ion transporters in *B. juncea*, a cation-efflux family gene, *BjCET1*, was cloned and functionally characterized.

**Results:**

BjCET1 contains 382 amino acid residues, including a signature motif of the cation diffusion facilitator protein family, six classic trans-membrane-spanning structures and a cation-efflux domain. A phylogenetic analysis showed that BjCET1 has a high similarity level with metal tolerance proteins from other *Brassica* plants, indicating that this protein family is highly conserved in *Brassica*. *BjCET1* expression significantly increased at very early stages during both cadmium and zinc treatments. Green fluorescence detection in transgenic tobacco leaves revealed that BjCET1 is a plasma membrane-localized protein. The heterologous expression of BjCET1 in a yeast mutant increased the heavy-metal tolerance and decreased the cadmium or zinc accumulations in yeast cells, suggesting that BjCET1 is a metal ion transporter. The constitutive expression of BjCET1 rescued the heavy-metal tolerance capability of transgenic tobacco plants.

**Conclusions:**

The data suggest that BjCET1 is a membrane-localized efflux transporter that plays essential roles in heavy metal ion homeostasis and hyper-accumulation.

**Supplementary Information:**

The online version contains supplementary material available at 10.1186/s12870-022-03569-x.

## Background

Several heavy metal ions, such as zinc (Zn^2+^) and cobalt (Co^2+^), are essential trace elements involved in various vital biological processes [1]. However, large amounts of heavy metal ions, even essential ones, can inactivate functional proteins and block biological processes [2]. In addition, for the non-essential elements, such as cadmium (Cd^2+^), accumulation even at low concentrations can cause toxicity [3]. To cope with these challenges, plants have thus evolved a complex network for metal ion uptake, trafficking, storage and efflux [4, 5].

Many Brassicaceae plants have particular mechanisms for heavy metal ion detoxification and homeostasis [6–8]. Several cation transporter families are involved in metal transport and storage, such as cation diffusion facilitator (CDF) proteins, natural resistance-associated macrophage proteins, yellow-stripe 1-like proteins, zinc-regulated transporter and iron-related transporter proteins, and cation exchangers [9, 10].

In plants, CDF family members, also known as metal tolerance proteins (MTPs), are vacuole membrane-localized and heavy-metal tolerance-related proteins [11–13]. On the basis of phylogeny, the CDF family proteins are classified into three clusters: Zn-, iron (Fe)- and manganese (Mn)-CDFs [14]. The typical structure of a CDF protein consists of six transmembrane domains, one modified CDF signature and one C-terminal cation efflux domain [15]. The model plant *Arabidopsis thaliana* possesses 12 MTPs, and the functions of several AtMTPs have been well-studied. For example, AtMTP1 has a cytosolic histidine-rich loop and is involved in sensing cytosolic Zn^2+^ [16]. AtMTP3 maintains metal ion homeostasis by regulating the exclusion of Zn^2+^ from the shoots to the roots [17]. AtMTP5 forms a complex with AtMTP12 to transport Zn^2+^ from the cytoplasm to Golgi apparatus [18]. AtMTP11 is an Mn transporter that confers Mn tolerance [19].

Phytoremediation is an ecologically and economically sound strategy to eliminate heavy metal ions from contaminated soils [20]. *Brassica juncea* is frequently used for the remediation of soils contaminated with heavy metals, owing to its high ability to fix metal ions in aboveground plant parts [21, 22]. Several metal transporters have been identified in *B. juncea*. BjYSL7 encodes a plasma-localized transporter that is involved in the transport of Fe^2+^, Cd^2+^ and nickel (Ni^2+^) ions from roots to shoots [23]. BjHMA4R, a heavy metal efflux pump, specifically binds to Cd^2+^ in the cytosol at low concentrations [24]. The bZIP transcription factor BjCdR15 is a regulator of Cd^2+^ uptake and transport in shoots [25]. Thus, the identification and functional analyses of *B. juncea* CDF family proteins are important for designing and breeding metal-accumulating plants.

Previously, four cation-efflux transporter-encoding genes (*BjCET1–4*) were predicted in *B. juncea*, and functions for *BjCET2*, *− 3* and *− 4* in the regulation of ion homeostasis have been reported [25, 26]. However, the biological function of *BjCET1* is largely unknown. Here, the contribution of BjCET1 to heavy-metal tolerance, as well as its potential role in the phytoremediation of heavy metal-contaminated soils, were investigated.

## Results

### Sequence analysis of the *BjCET1* gene

**Fig. 1:**
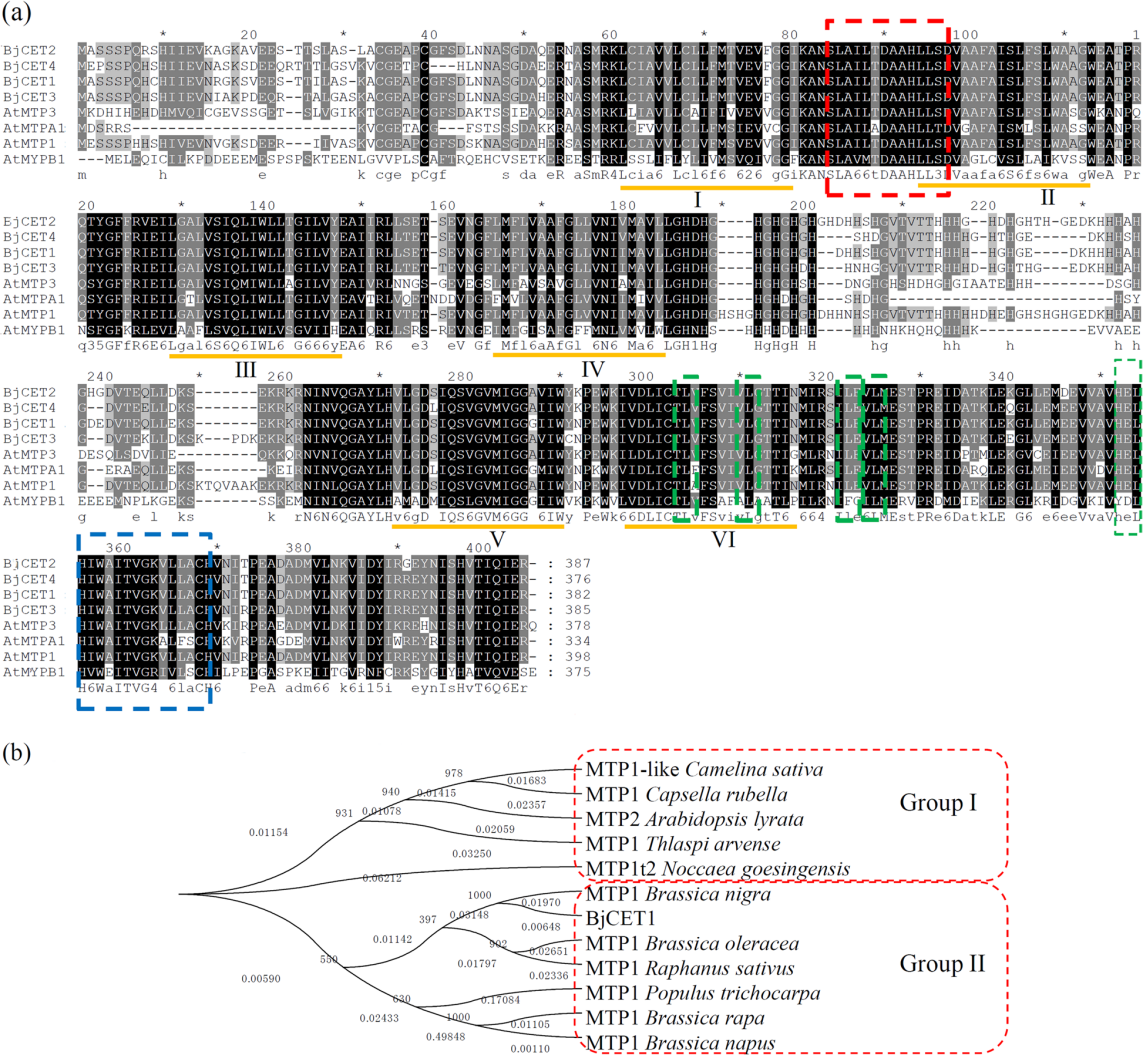
Basic sequence analysis of BjCET1. **a** Sequence alignments of predicted amino acid sequences for BjCET1-4, AtMTP1, AtMTP3, AtMTPA1, and AtMTPB1 was performed by CLUSTALW. The classic protein features are highlighted: six transmembrane domains are underlined in dark yellow; the CDF signature is marked by red dashed box; the C-terminal LZ motifs are marked by green dashed boxes; and the C-terminal putative Zn binding site HD(E)XHXWXL(I)TX_8_H is marked by blue dashed box. **b** Phylogenetic analysis of the reported CDF/MTP proteins from different plant species. The Neighbor–Joining phylogenetic tree was constructed using MEGA6.1 after CLUSTALW alignment of the full-length amino acid sequences

According to the previously published sequence information (GenBank ID: AY187082), the full-length cDNA *BjCET1* was cloned [27]. BjCET1 contains a putative open read frame of 1146 bp encoding 382 amino acid residues. The putative BjCET1 protein possesses a CDF family signature motif (SLAILTDAAHLLSD) at the *N*-terminus, six classic trans-membrane-spanning structures in the middle region, and a cation-efflux domain at the *C*-terminus (Fig. 1a).

A phylogenetic analysis showed that BjCET1 has high similarity with other well-identified MTP proteins (Fig. 1b). Most of the selected proteins were classed into Groups I or II, and all the MTPs from the *Brassica* family, such as *B. nigra*, *B. oleracea*, *B. rapa* and *B. napus*, were placed into the same group (Group II). Our data indicated that MTP/CDF proteins are highly conserved in *Brassica*. BjCET1 and MTP1 from *B. nigra* were grouped into one gene pair in the evolutionary tree, suggesting similar biological functions.

### Expression analysis of *BjCET1* under different heavy-metal treatments

To investigate the basic biological function of *BjCET1*, a tissue-specific expression analysis was performed. The *BjCET1* gene was expressed highest in the roots and lowest in the leaves (Additional file 1).

**Fig. 2:**
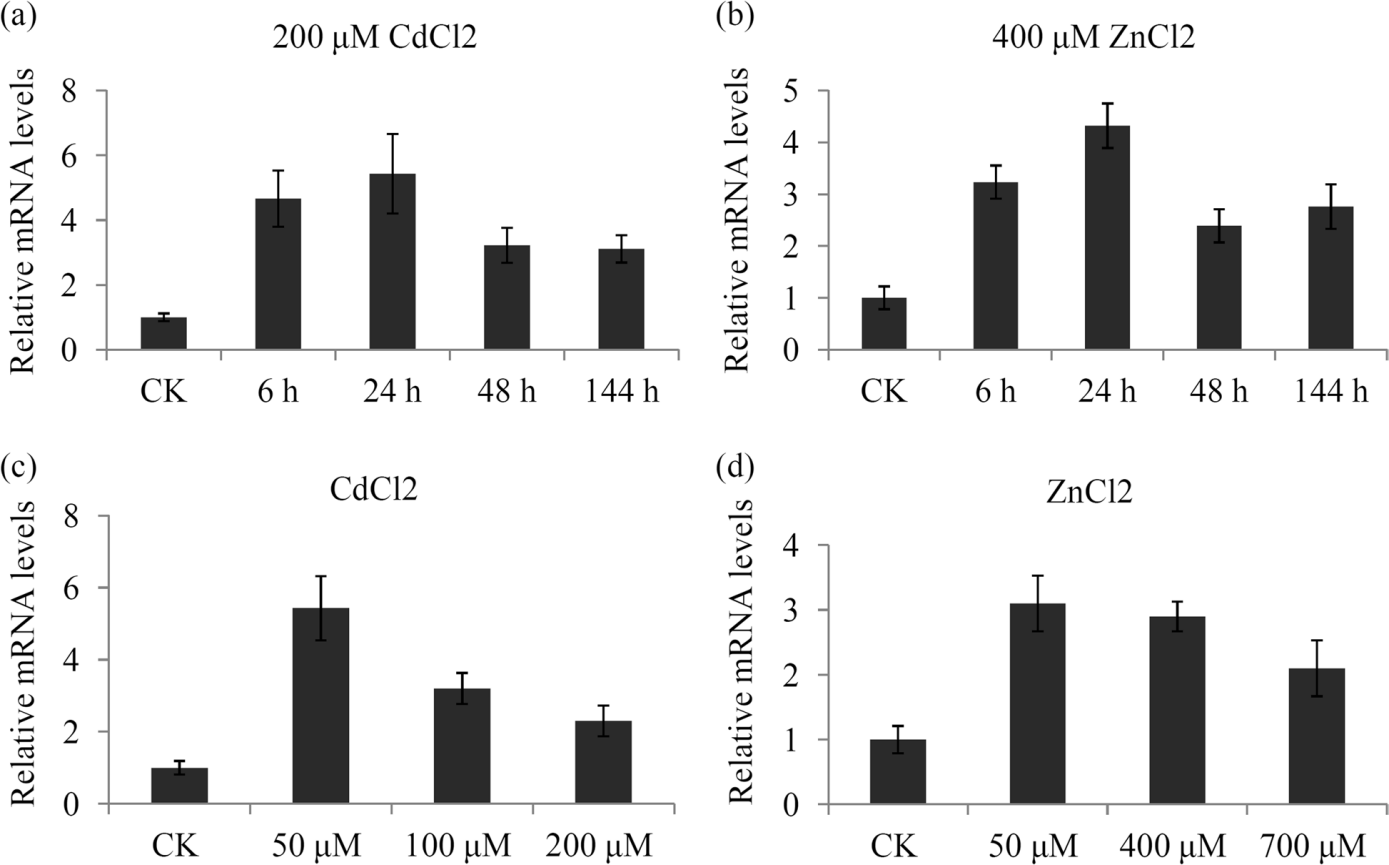
Expression analysis of *BjCET1* under heavy metal treatments. **a** The relative mRNA levels of *BjCET1* gene under 200 μM CdCl_2_ treatment. **b** The relative mRNA levels of *BjCET1* gene under 400 μM ZnCl_2_ treatment. **c** The relative mRNA levels of *BjCET1* under CdCl_2_ treatments at different concentrations. **d** The relative mRNA levels of *BjCET1* under ZnCl_2_ treatments at different concentrations. Data are expressed as means±SD of three biological repeats. “*” indicates significant differences in relative mRNA levels of *BjCET1* gene

To reveal the *BjCET1* expression pattern in response to different heavy-metal stresses, qRT-PCR was performed. Under CdCl_2_ or ZnCl_2_ treatment, the expression of *BjCET1* was significantly up-regulated at early stages and peaked at 24 h (Fig. 2a and b). *BjCET1* expression was induced by CdCl_2_ at all concentrations tested. The expression level of *BjCET1* under at high CdCl_2_ concentrations (100 and 200 μM) was lower than at the low CdCl_2_ concentration (50 μM) (Fig. 2c). Compared with the control, the *BjCET1* expression largely increased during ZnCl_2_ treatments, and no significant differences were observed between different ZnCl_2_ concentrations (Fig. 2d).

### Subcellular localization of the BjCET1 protein

**Fig. 3:**
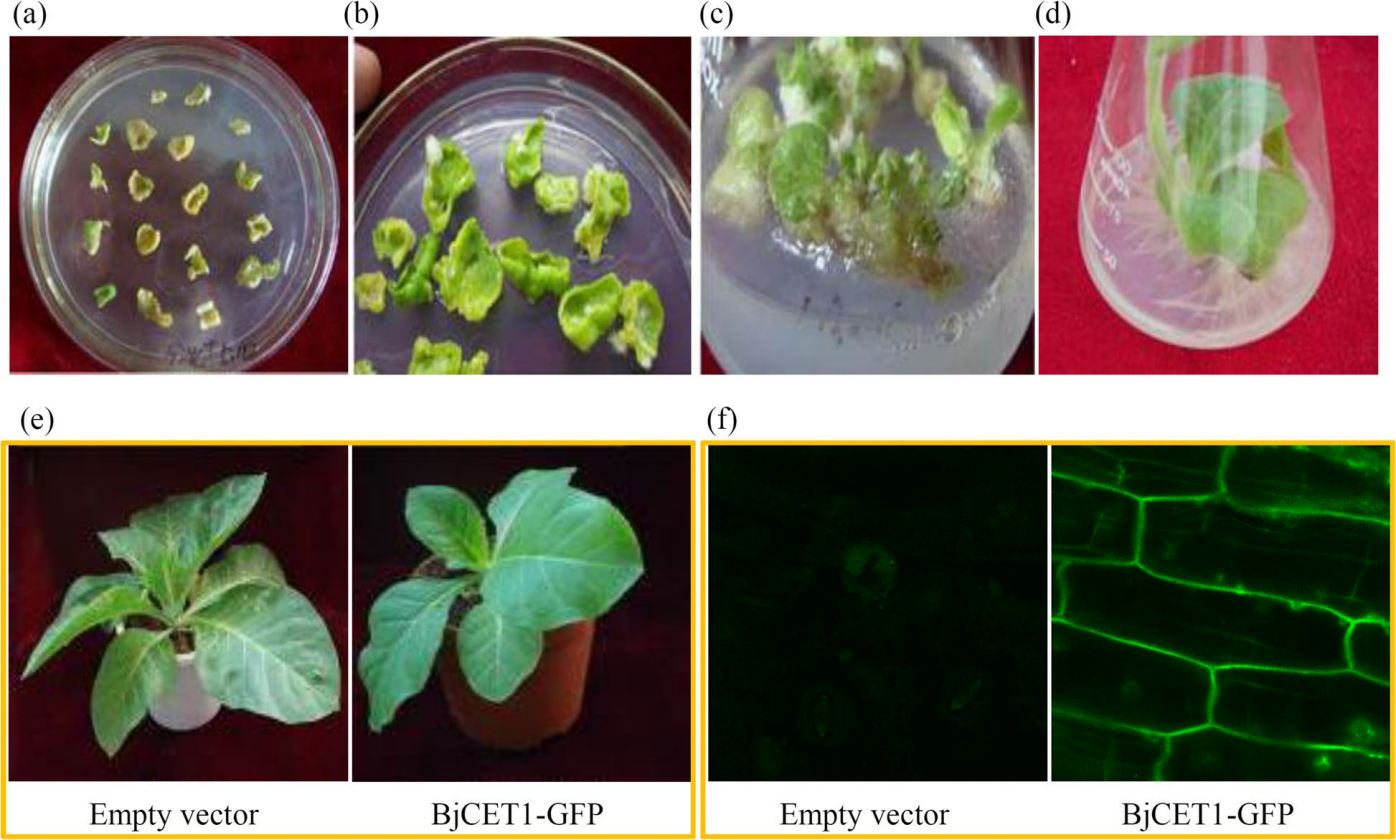
Genetic transformation and subcellular localization of BjCET1 in tobaccos. **a**-**e** The growth process of tobacco after *Agrobacterium* mediated transformation. **f** Subcellular localization of BjCET1 protein in root cells of transgenic tobaccos. Fluorescence of GFP was observed by using confocal laser scanning microscopy under 485 nm and 364 nm

The resistant tobacco plants harboring pBIN-BjCET1-mGFP5-ER and pBIN-mGFP5-ER vectors were obtained through *Agrobacterium tumefaciens*-mediated leaf-disc transformation. The process from calli differentiation to seedling growth is shown in Fig. 3a-d. The genomic DNAs of WT and transgenic tobacco plants were extracted, and the positive transgenic plants were identified using PCR assay. The target PCR product (950 bp) was detected in the transgenic plants, indicating that the T-region of the expression vector was integrated into the tobacco genome. The BjCET1 protein was found to be localized at the plasma membrane, suggesting that BjCET1 might be a potential transporter (Fig. 3e-f).

### Involvement of BjCET1 in broad-range metal stress tolerance

**Fig. 4:**
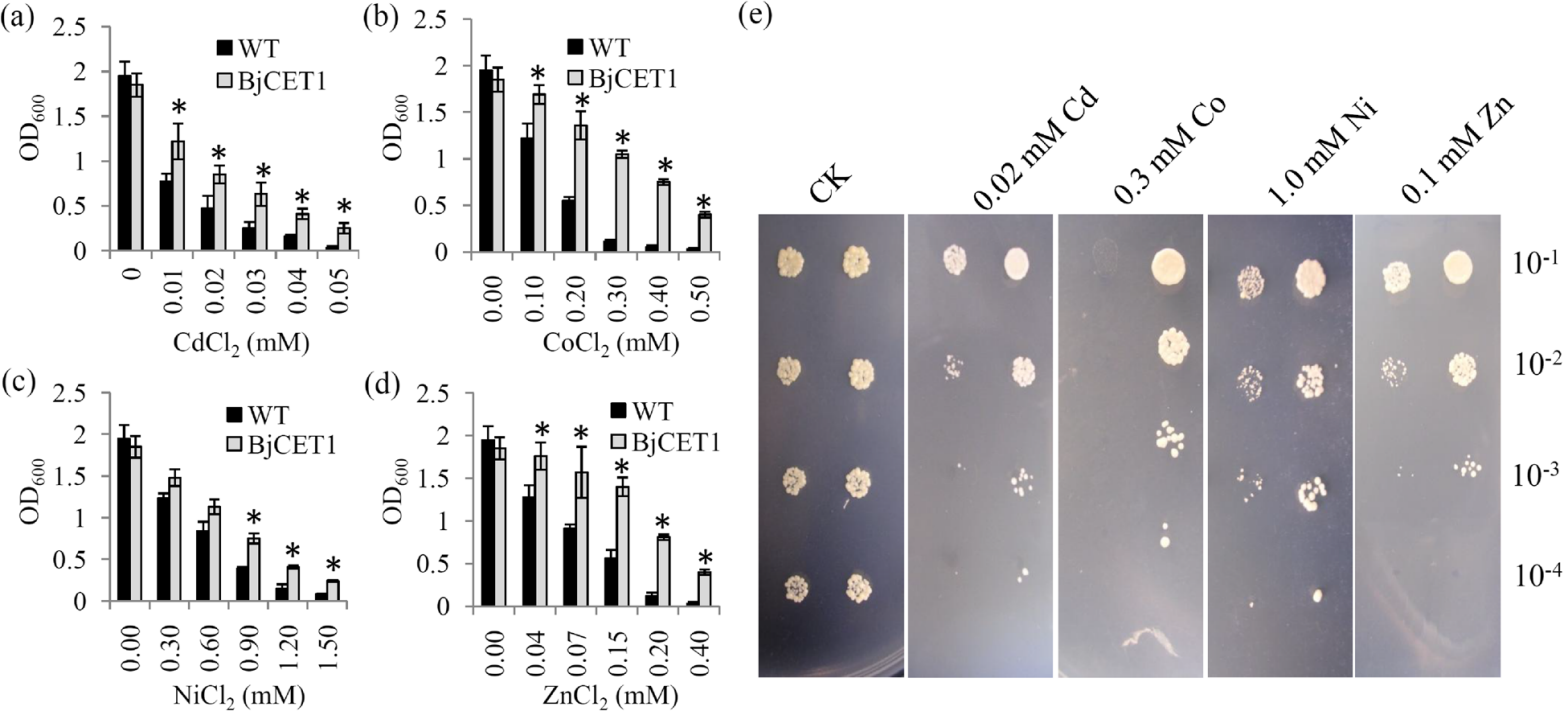
Involvement of BjCET1 in a broad range of metal stress tolerance. **a** The growth status of yeast strains expressing BjCET1 under CdCl_2_ treatment at different concentrations. **b** The growth status of yeast strains expressing BjCET1 under different concentrations of CoCl_2_ stresses. **c** The growth status of yeast strains expressing BjCET1 under different concentrations of NiCl_2_ stresses. **d** The growth status of yeast strains expressing BjCET1 under different concentrations of ZnCl_2_ stresses. **e** Growth of yeast YK44 cells expressing BjCET1 and the pYES2 empty-vector control cells in YPGAL plates for 48–72 **h**. The YPGAL plates contained 0.02 mM Cd, 0.3 mM Co, 1.0 mM Ni, or 0.1 mM Zn

To determine its functional properties and substrate specificities, BjCET1 was heterologously expressed in the yeast *cot1&zrc1* (YK44) deficient mutant. Yeast cells containing empty pYES2 vector served as controls. Yeast cells expressing the BjCET1 protein were exposed independently to CoCl_2_, NiCl_2_, ZnCl_2_ or CdCl_2_ at different concentrations. The over-expression of BjCET1 enhanced the viability of transgenic yeasts against Co^2+^, Ni^2+^, Zn^2+^ and Cd^2+^ treatments (Fig. 4a-d).

To confirm the role of BjCET1 in heavy-metal tolerances, the growth states of yeast cells expressing the BjCET1 protein was observed on solid medium. The growth rates of both control and transgenic yeast cells were slower under heavy metal stress than under the control conditions. However, the growth capabilities of transgenic yeast under Ni^2+^, Co^2+^, Zn^2+^ and Cd^2+^ stresses were significantly greater than under control conditions (Fig. 4e). The data suggest that BjCET1 improved the resistances of yeast cells to various heavy metal ions.

### The metal ion transport activity of BjCET1

**Fig. 5:**
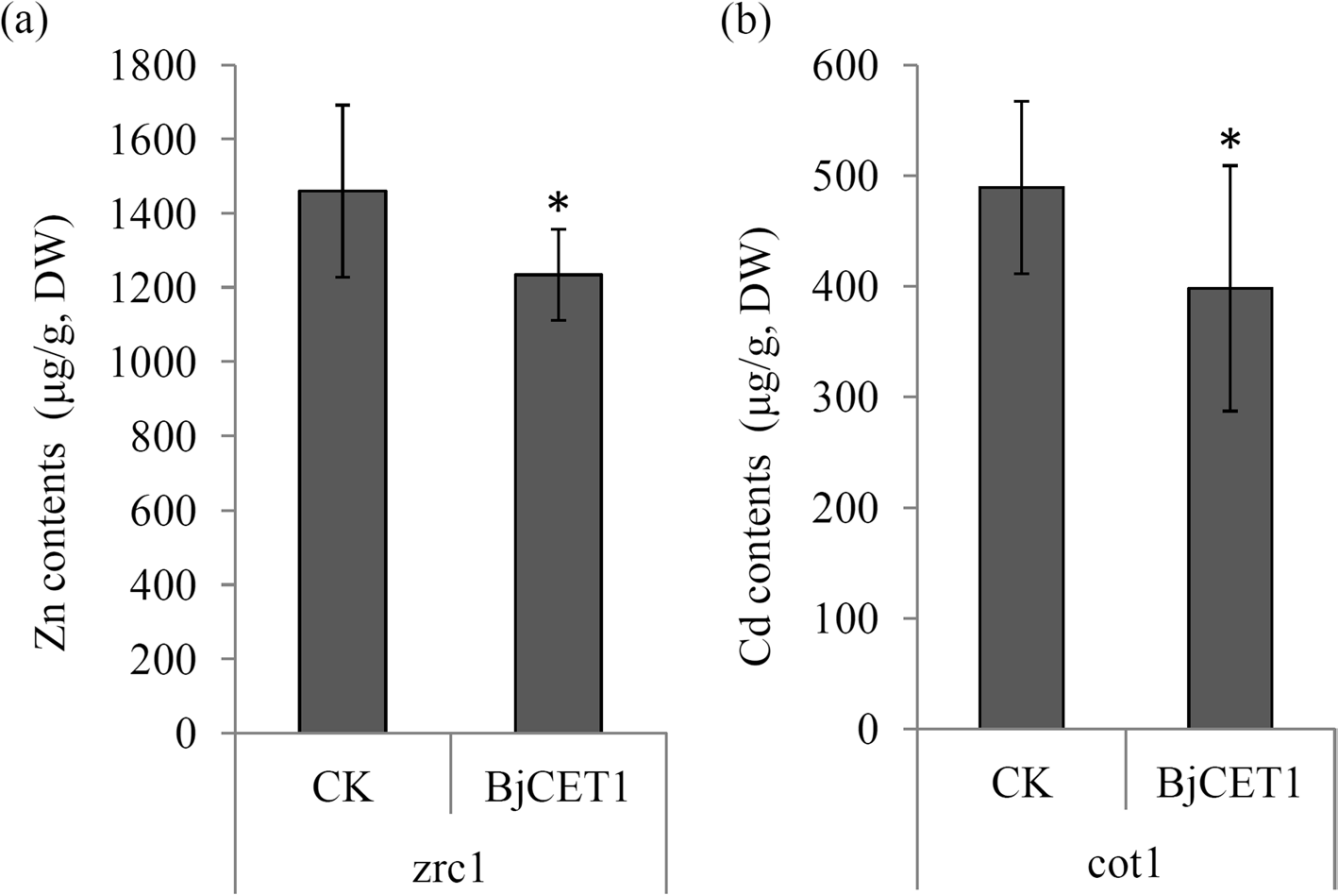
Metal ion transport activity of BjCET1. **a** ICP-MS assay of Zn content in zrc1 yeast cells. The yeast cells were incubated in 125 μM ZnCl_2_ for 4 h before collection. **b** ICP-MS assay of Cd content in *cot1* yeast cells. The yeast cells were incubated in 125 μM CdCl_2_ for 4 h before collection. A significant change in Zn or Cd accumulation was detected in *BjCET1* expressing yeasts compared to control yeast (*P* < 0.01)

The metal ion transport activity of BjCET1 was determined using the ICP-MS method. Compared with the control, the yeast *zrc1* mutant over-expressing BjCET1 exhibited a reduced Zn accumulation, from 1460 μg/g to 1234 μg/g upon the ZnCl_2_ treatment (Fig. 5a). Compared with the control, the yeast *cot1* mutant over-expressing BjCET1 also exhibited a reduced Cd accumulation, from 489 μg/g to 398 μg/g upon the CdCl_2_ treatment (Fig. 5b). The data suggest that BjCET1 might be a metal ion transporter that exports Zn and Cd ions out of yeast cells.

### Heavy-metal tolerance of *BjCET1-*transformed tobacco

**Fig. 6:**
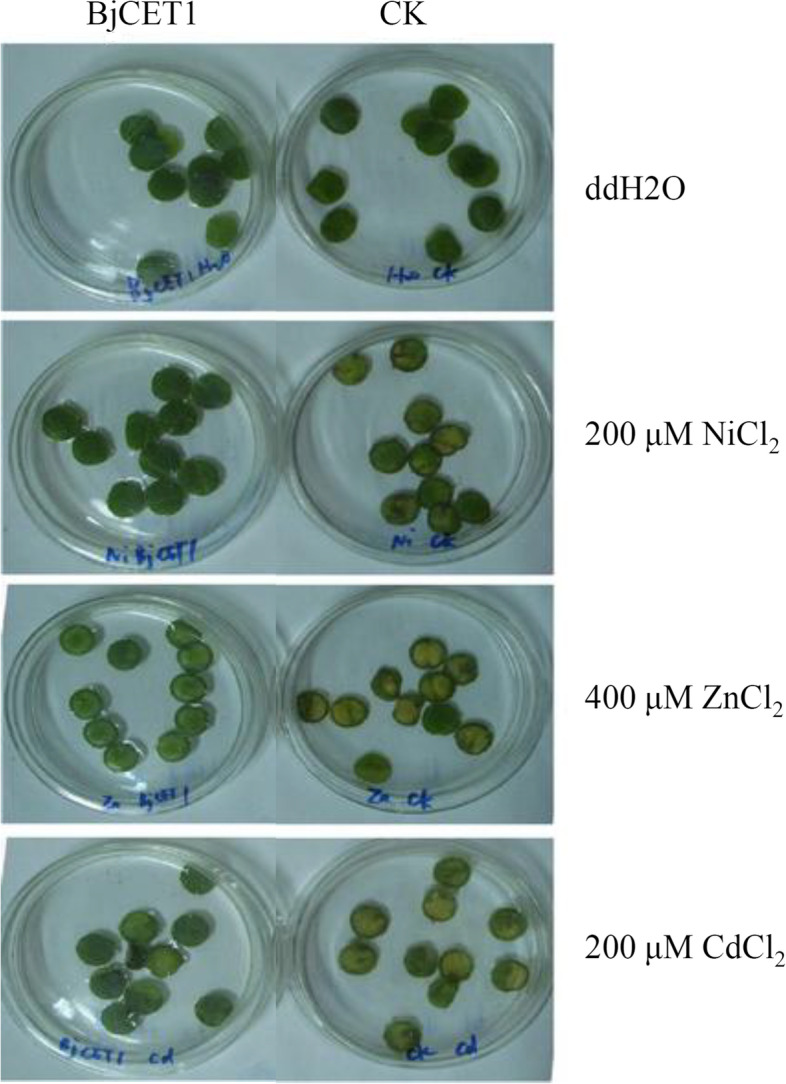
Heavy metal tolerance of BjCET1 transgenic tobacco leaves. The healthy transgenic tobacco leaves were selected as control. The round blades were placed in ddH_2_O, 200 μM NiCl_2_, 400 μM ZnCl_2_, or 200 μM CdCl_2_. Ten independent pieces were placed in each Petri dish. The status of the leaves was observed after 5 days

To investigate the role of *BjCET1* in plants, the *BjCET1* gene was heterologously over-expressed in tobacco seedlings. The leaves from *BjCET1* over-expressing tobacco seedlings and WT were placed in ddH_2_O, 200 μM NiCl_2_, 400 μM ZnCl_2_ or 200 μM CdCl_2_ solution. There were no obvious differences between the transgenic and WT tobacco leaves in the ddH_2_O solution. However, under various heavy-metal stresses, the WT leaves turned yellow and rotted, while the transgenic leaves remained green (Fig. 6). The damage on transgenic tobacco leaves was significantly lower than on WT leaves, suggesting that over-expressing *BjCET1* greatly improved tobacco resistance to heavy-metal stresses.

### Heterologous expression of BjCET1 enhanced tobacco’s heavy metal tolerance

**Fig. 7:**
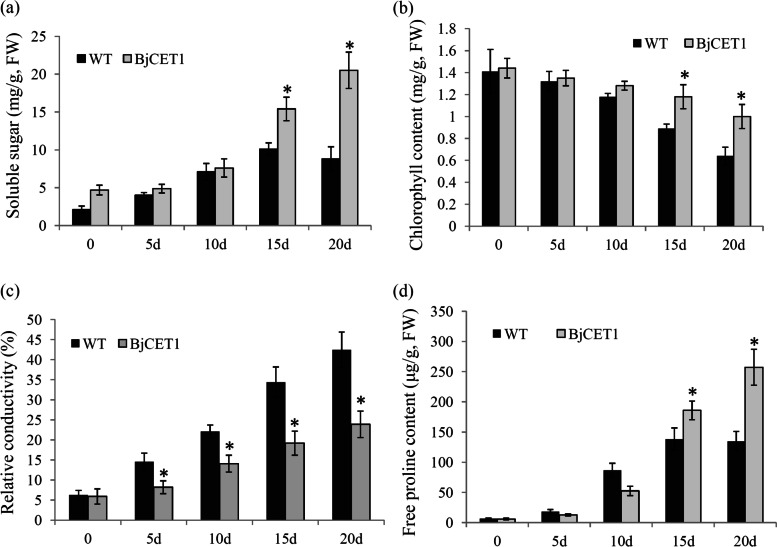
Physiological and biochemical changes of tobacco during Cd treatment process. Several physiological parameters, including soluble sugar (**a**), chlorophyll content (**b**), relative conductivity (**c**), and free proline content (**d**), were analyzed under the CdCl_2_ treatment. A *P* value < 0.01 was considered to be statistically significant and indicated by “*”

Tobacco seedlings independently over-expressing BjCET1 and the empty vector were selected to analyze the roles of BjCET1 in Cd tolerance. Four important physiological parameters, including relative conductivity, soluble sugar content, chlorophyll content and free proline content, were determined in the transgenic and WT tobacco leaves. The soluble sugar contents were largely up-regulated by the CdCl_2_ treatment at different time points. Compared with WT, the soluble sugar contents in the BjCET1 transgenic plants were significantly higher at time points 15 d and 20 d (Fig. 7a). The chlorophyll contents were obviously reduced by the CdCl_2_ treatment. At time points 15 d and 20 d, the chlorophyll contents in the BjCET1 transgenic tobacco were higher than in WT (Fig. 7b). The relative conductivities were also greatly increased during the CdCl_2_ treatment. Under the CdCl_2_ treatment, the relative conductivity levels in the bjCET1 transgenic tobacco were lower than that in WT (Fig. 7c). Compared with WT, the free proline contents in the BjCET1 transgenic plants were significantly higher than in WT at time points 15 d and 20 d (Fig. 7d).

## Discussion

Higher plants possess a complex system for heavy metal ion uptake, absorption, transport, and storage, allowing them to cope with various heavy-metal stresses [28]. *B. juncea* is a rapid-growth plant with an appreciable capacity to absorb various toxic heavy metals. Thus, *B. juncea* is frequently applied to the ‘green’ remediation of toxic metal-contaminated mining soils [29].

The CDF/MTP family plays significant roles in maintaining intracellular ion homeostasis and tolerance in plants [30]. To date, several CDF/MTP family genes of *B. juncea* have been cloned and functionally characterized, such as *BjCET2*, *3*, and *4* [26, 31]. In the present study, the *BjCET1* gene was cloned and functionally characterized, giving us an opportunity to understand the entire CDF/MTP family in *B. juncea*.

Most of the CDF/MTPs contain six transmembrane domains, a cytoplasmic N-terminus and a characteristic C-terminal cation efflux domain [14]. Furthermore, a Leu zipper motif at the C-terminus of CDF/MTP family members is highly conserved and critical for Zn detoxification [32]. Similar to the previously published characterization of BjCET proteins, BjCET1 possesses a classic Zn-CDF signature, indicating that BjCET1 is a heavy-metal transporter. BjCET2 and BjCET4 are mainly expressed in roots, and BjCET3 is constitutively expressed in all tested tissues [26, 31]. Similar expression patterns reflect the shared functions of BjCET1, − 2 and − 4. The root-specific expression of BjCET1 further suggested that it functions in the root system against heavy metal over-accumulation.

In plants, the expression levels of many CDF/MTP family genes are induced in different tissues during heavy-metal treatments. For example, the expression of *OsMTP11* was substantially enhanced at 4 h after exposure to Cd, Zn, Ni and Mn treatments [33]. In *Populus trichocarpa*, Cd exposure significantly enhances the expression of *MTP11.1*, and Zn exposure significantly increases the expression of *MTP10.3* [34]. The expression level of *BjCET1* was significantly increased during both Cd and Zn treatments, indicating an important role of *BjCET1* in responses to heavy-metal stresses. Interestingly, *BjCET1* was significantly induced at 6 h, suggesting that *BjCET1* is a rapid-response gene.

CDF/MTP family members are responsible for the transport of heavy metal ions, such as Zn^2+^, Co^2+^, Ni^2+^ and Cd^2+^, in plants [34, 35]. The heterologous expression of *CDF/MTP* genes in yeast deficiency mutants is a good method to reveal their roles in heavy-metal tolerance [11]. The heterologous expression of *B. napus MTP3* in yeast mutant cells enhances tolerance to, and intracellular sequestration of, Zn^2+^ and Mn^2+^ [30]. The heterologous expression of *Camellia sinensis MTP8.2* in yeast cells confers tolerance to Ni^2+^ and Mn^2+^ but not to Zn^2+^ [13]. The heterologous expression of rice *MTP1* in tobacco relieves Cd stress-induced phototoxic effects [36], and the heterologous expression of *OsMTP1* in the yeast *zrc1cot1* complements the Zn^2+^ and Co^2+^ hypersensitivities of this mutant [37]. In our study, the growth rate of the control yeast mutant was lower than that of the transgenic yeast under various heavy-metal stresses, suggesting that the heterologous expression of *BjCET1* created a broad-range metal-stress tolerance in YK44 yeast cells.

The ICP-MS data suggested a potential metal ion transport activity for BjCET1 in yeast cells. To confirm the biological function of BjCET1 in plants, it was heterologously expressed in tobacco plants. In BjCET1-GFP transgenic tobacco plant leaves, the fusion protein mainly was found to be localized at the cell membranes. The CDF family proteins may be efflux transporters that are involved in metal homeostasis by maintaining optimal metal concentrations in the cytoplasm [12, 38]. The constitutive expression of BjCET1 greatly rescued the heavy metal-tolerance capabilities of transgenic tobacco plants. The data suggested that BjCET1 is a membrane-localized efflux transporter that plays important roles in heavy metal ion homeostasis and hyper-accumulation [39].

## Conclusion

In our study, a heavy metal transporter-encoding gene, *BjCET1*, was cloned and functionally characterized. A sequence analysis showed that BjCET1 contained a classic Zn-CDF signature and was highly similar to MTP1 from *B. nigra*. The expression of *BjCET1* was rapidly up-regulated under various heavy metal ion treatments. Yeast experiments suggested that BjCET1 is involved in a broad-range metal stress tolerance. Furthermore, the heterologous expression of BjCET1 enhanced the heavy-metal tolerance of tobacco. BjCET1 has great potential in the phytoremediation of heavy metal-contaminated environments.

## Methods

### Plant materials

*B. juncea* materials were obtained from the North Central Regional Plant introduction station of the United States National Plant Germplasm System (order number: IP173874). *Nicotiana tabacum* Cv. W38 was used for heterologous expression assay. All plant materials were grown routinely at room temperature in a greenhouse at Hangzhou Normal University. Fourteen-day-old seedlings were selected and treated with heavy metal ions at various concentrations.

### Isolation of the *BjCET1* gene

Total RNAs of *B. juncea* seedlings were extracted using TRIzol reagent (Invitrogen, Shanghai, China) in accordance with the manufacturer’s protocol. RNAs were used as templates for the first- and second-strands cDNA synthesis using a cDNA preparation kit (Illumina, San Diego, CA, USA). Using cDNA as the template, the cDNA sequence of *BjCET1* was cloned by PCR amplification. The primer sequences are listed in Additional file 2.

### Bioinformatic analysis of BjCET1 protein

The BjCET1 full-length protein sequence and other CET family members from *B. juncea* and MTP proteins from Arabidopsis were used for multiple sequence alignments. The alignments were performed using ClustalW with default parameters and were visualized subsequently using GeneDoc software (http://www.nrbsc.org/gfx/genedoc/). An unrooted phylogenetic tree of CET family proteins was constructed using MEGA6.1 (http://www.megasoftware.net/) employing the neighbor-joining method.

### Expression analysis of *BjCET1*

Up- to the 5-6 leaf stage, the seedlings were used for tissue-specific expression analysis. Three major tissues, including root, leaf and shoot, were used in tissue-specific expression analysis experiment. For stress treatments, seedlings were hydroponically planted in aerated liquid medium containing CdCl_2_ or ZnCl_2_. Several previous studies have focused on the responses of *B. juncea* to Cd treatment. In Shu’s study, 50 μM CdCl_2_ was used to treat *B. juncea* seedlings; In Bhardwaj’s study, 200, 400, and 600 μM CdCl_2_ were used to treat *B. juncea* seedlings; 50 μM of CdCl_2_ was used to treat *B. juncea* seedlings; In Misra’s study, 10-160 μM of CdCl_2_ were used to treat *B. juncea* seedlings [35, 40–42]. Thus, 0, 50, 100, 200 μM of CdCl_2_ were used in our study. For ZnCl_2_ treatment, the concentration of ZnCl2 was set according to Lang’ s study [26].

The seedlings of 5-6 leaf stage were subjected to 200 μM CdCl_2_ or 400 μM ZnCl_2_ solutions for 6 h, 24 h, 48 h, or 144 h. After treatment, leaves were collected and washed with ddH_2_O and quickly kept in liquid nitrogen until used. The primer sequences for the qRT-PCR are listed in Additional file 2.

QRT-PCR was performed using a SYBR Premix Ex Taq Kit (TaKaRa, Dalian, China) on an ABI PRISM 7700 DNA Sequence Detection System (Applied Biosystems, Shanghai, China). The ACTIN gene of *B. juncea* was used as an internal reference (5#-AAGATCTGGCATCACACTTTC-3# and 5#-TAGTCAACAGCAACAAAGGAG-3#) for the relative quantification of transcript levels [26]. The relative fold differences in the *BjCET1* gene were calculated by the values of comparative cycle threshold (2^-ΔΔCt^). In detail, 1 μL cDNA sample and 0.1 μM of each primer were added to 5 μL of 2 × SYBR Green solution [43]. Five technical repetitions and three biological replicates were performed in qRT-PCR experiments.

### Yeast experiments for the metal-tolerance assay

The cDNA of *BjCET1* was cloned into the pYES2 vector. The BjCET1-pYES2 construct and empty pYES2 vector were transformed independently into the YK44 *Saccharomyces cerevisiae* mutant strain (*ura3-52*, *his3-200*, *zrc1*, *cot1*, *MATα*). The transformed yeast mutant cells were selected on solid medium containing SMM-uracil, 6.7 g yeast nitrogen base without amino acids, 20 mg histidine, 2% glucose and 2% agar. The presence of the *BjCET1* insert sequence in the selected yeast cells was confirmed by PCR amplification.

The metal-tolerance experiment was performed in accordance with a previously published protocol [26]. In detail, yeast cells were grown in 10 mL of SMM-uracil liquid medium until reaching OD_600_ = 1.5. Then, 1 mL of yeast culture solution was transferred to a 50 mL of SMM-uracil liquid medium. The mixed culture was poured into agar dishes and allowed to cool. Solutions containing Cd^2+^ or Zn^2+^ ions were spread onto the agar, and the plates were maintained in an incubator at 30 °C for 3 days.

### Yeast experiments for the metal-ion accumulation assay

To investigate the efflux transport activity of the BjCET1 protein, the accumulation of Zn^2+^ (or Cd^2+^) in selected yeast cells treated with ZnCl_2_ (or CdCl_2_) was analyzed. With ZnCl_2_ (or CdCl_2_) supplements, *zrc1* (or *cot1*) yeast mutant cells containing the BjCET1 were added to 200 mL SMM–uracil liquid medium and cultured to OD_600_ = 0.2. The yeast cell culture was allowed to grow to OD_600_ = 0.4. Yeast cells were harvested by 5000×g centrifugation and washed three times with a 20-mM EDTA solution. The clean cells were dried and weighed. The Zn (or Cd) contents were analyzed using inductively coupled plasma-atomic emission spectrophotometry [26].

### Heterologous expression of the BjCET1:GFP fusion protein

The cDNA of *BjCET1* was inserted into the *Xba*I and *BamH*I sites of the pBI121-GFP vector (Additional file 3). The pBI121-BjCET1-GFP and pBI121 empty vectors were introduced independently into *Agrobacterium tumefaciens* (EHA105 strain) in accordance with a previously published method [44]. All the yeast transformants were selected on MS medium supplemented with 200 mg·L^− 1^ kanamycin and 250 mg·L^− 1^ Cef. Successful transformations were confirmed by PCR amplification. The primer sequences for the PCR are listed in Additional file 2. Tissue samples from the positive transgenic lines were used for the further experiments.

### Confocal microcopy analysis

The GFP fluorescence of the positive transgenic lines were visualized by confocal laser scanning microcopy (Zeiss, Jena, Germany). The parameters for the confocal microcopy were set as follows: 490 nm for excitation, 500 nm for cutoff, and 515 nm for emission.

### Heavy metal accumulation assay

Approximately 1 cm^2^ regions from the wild type (WT) and transgenic tobaccos were excised and placed in MS medium or MS medium containing 200 μM CdCl_2_. Each treatment group, containing 10 independent explants, was cultured at 25 °C. After 30 d, the plant samples were harvested and washed twice in ddH_2_O. To determine Cd contents, approximately 100-mg plant samples were digested with 2 mL HNO_3_ by heating discontinuously in a microwave for 2 min. The cooled solution was transferred to a 50-mL flask and ddH_2_O was added up to a standard volume before being analyzed using the ICP-MS method [26].

Four physiological parameters, soluble sugar, chlorophyll content, relative conductivity and free proline content, were determined in accordance with previously published methods [45].

## Supplementary Information

**Additional file 1: Figure S1**. Tissue-specific expression analysis of BjCET1 gene.

**Additional file 2: Table S1**. The detail information of the primer sequences.

**Additional file 3: Figure S2**. The vector map of pBI121-BjCET1-GFP.

**Additional file 4. Figure S3**. The full-length membranes of qRT-PCR gel. 

## Data Availability

All data generated or analysed during this study are included in this published article and its supplementary information files. The full-length membranes of qRT-PCR gel are showed in Additional file 4. No sequencing data was used in the present study.

## Abbreviations

CDF: Cation diffusion facilitator
MTP: Metal tolerance protein
PCR: Polymerase chain reaction
QRT-PCR: Quantitative reverse transcription PCR
WT: Wild type
