# Supplementary figures and images for: Identification and functional analysis of cation-efflux transporter 1 from Brassica juncea L
Source: BMC Plant Biol. 2022 Apr 6;22:174. doi: 10.1186/s12870-022-03569-x (PMC8985314; doi:10.1186/s12870-022-03569-x)

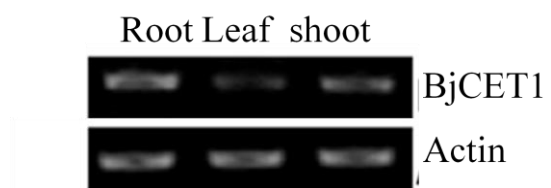

Figure S1 Tissue-specific expression analysis of BjCET1 gene.

Supplement: Supplementary file 1 — Additional file 1: Figure S1. Tissue-specific expression analysis of BjCET1 gene. [file 12870_2022_3569_MOESM1_ESM.pdf]

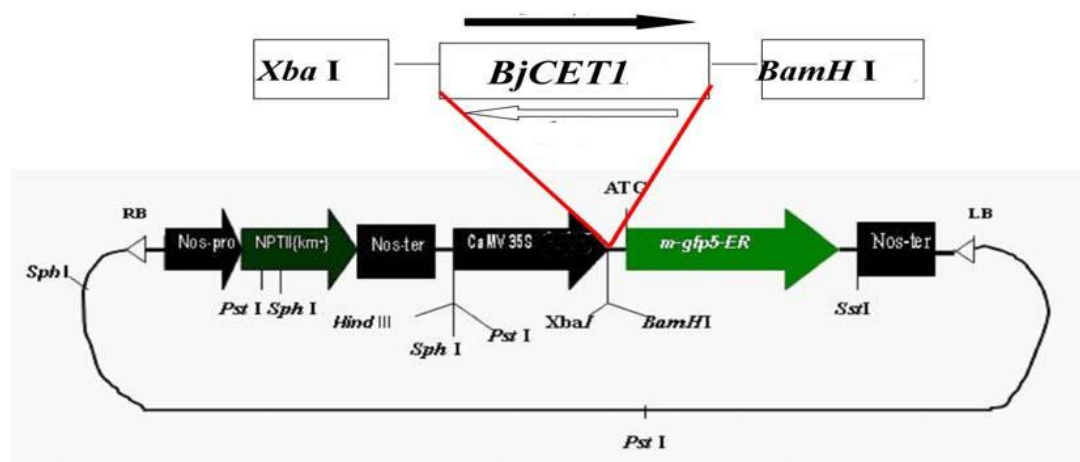

Figure S2 The vector map of pBI121-BjCET1-GFP.

Supplement: Supplementary file 3 — Additional file 3: Figure S2. The vector map of pBI121-BjCET1-GFP. [file 12870_2022_3569_MOESM3_ESM.pdf]
