# Supplementary material for: Identification and functional analysis of cation-efflux transporter 1 from Brassica juncea L
Source: BMC Plant Biol. 2022 Apr 6;22:174. doi: 10.1186/s12870-022-03569-x (PMC8985314; doi:10.1186/s12870-022-03569-x)
Supplement: Supplementary file 4 — Additional file 4. Figure S3. The full-length membranes of qRT-PCR gel. [file 12870_2022_3569_MOESM4_ESM.pptx]

## Slide 1
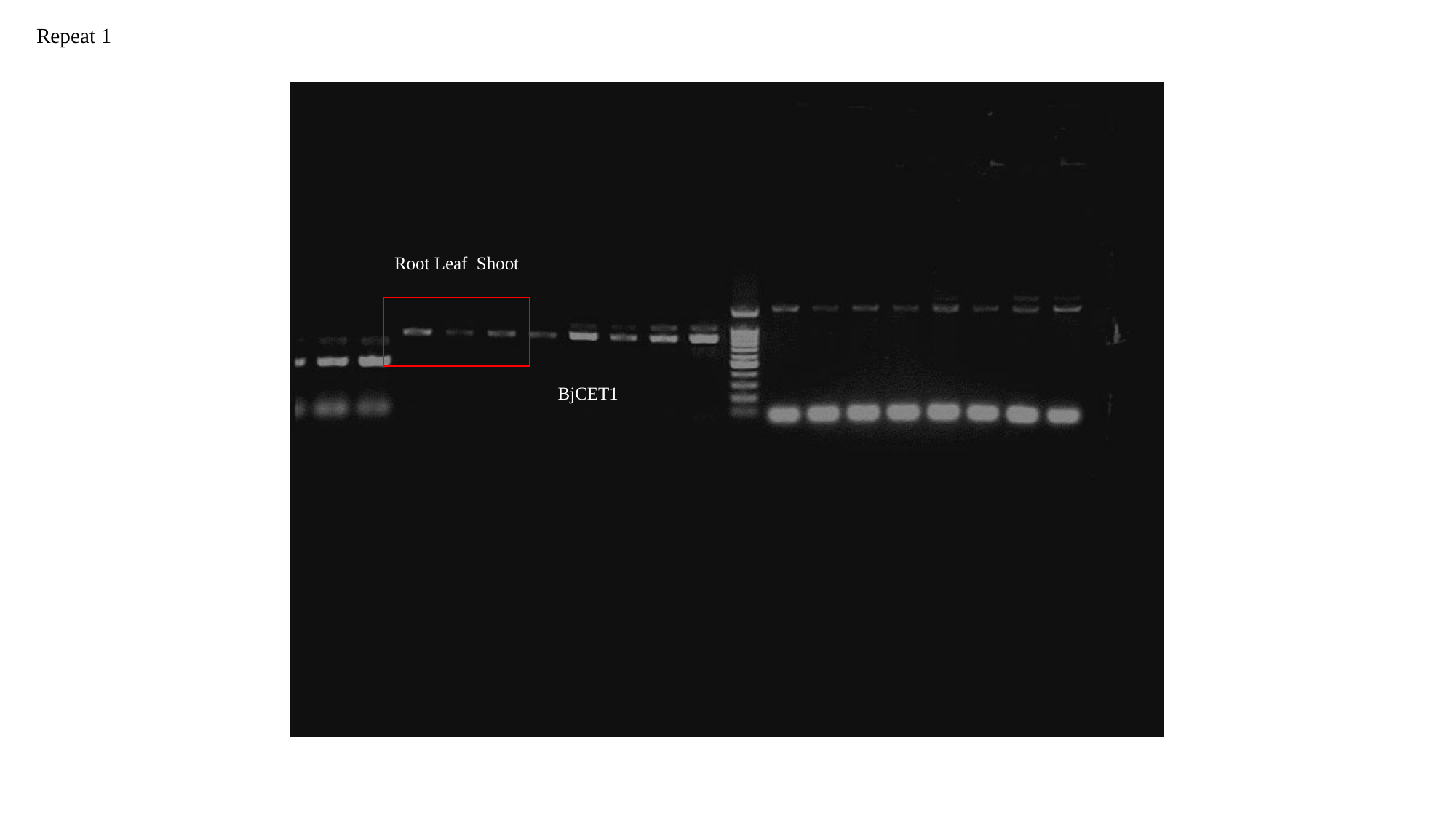

Repeat 1
Root Leaf Shoot
BjCET1

## Slide 2
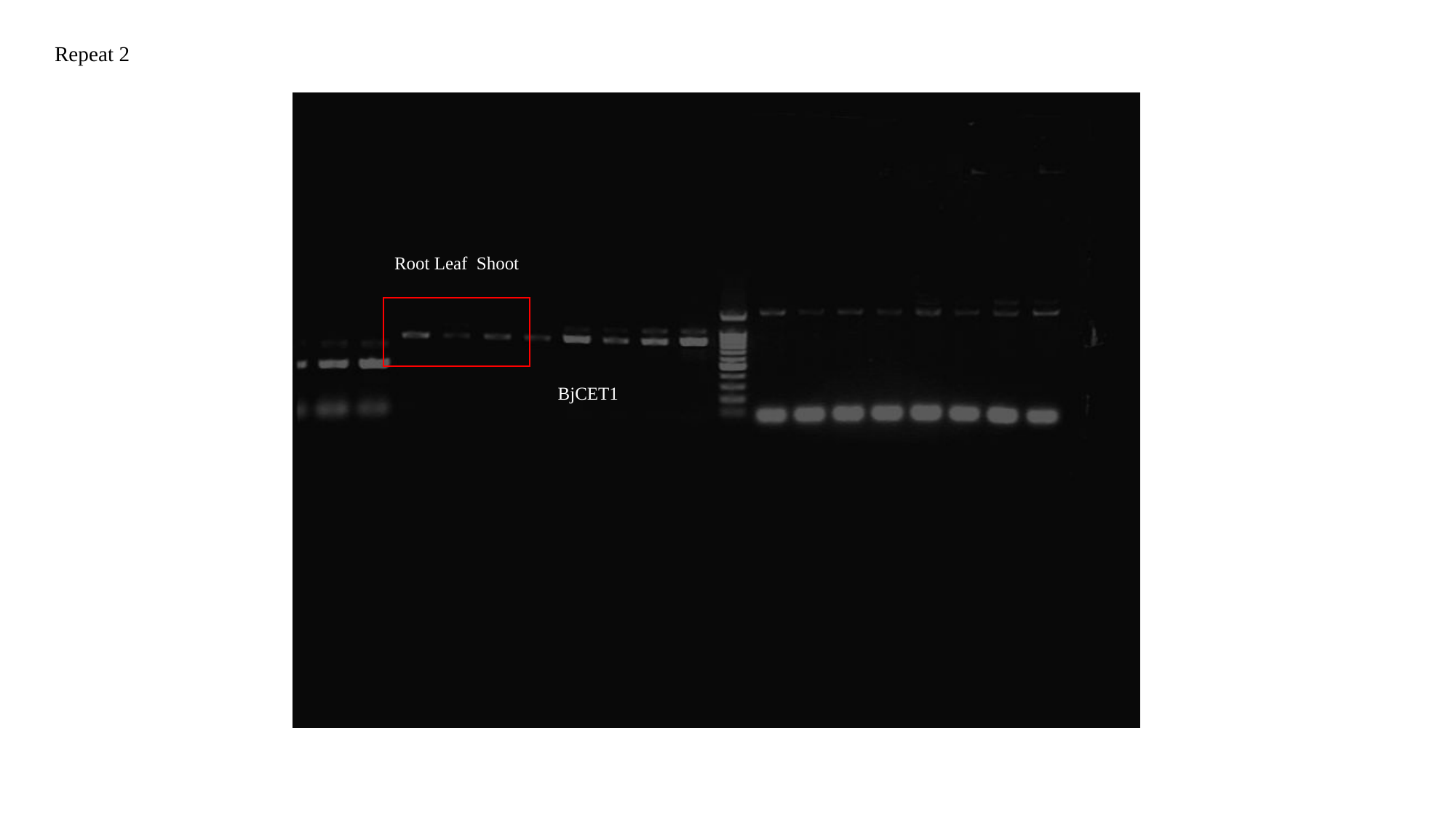

Repeat 2
Root Leaf Shoot
BjCET1

## Slide 3
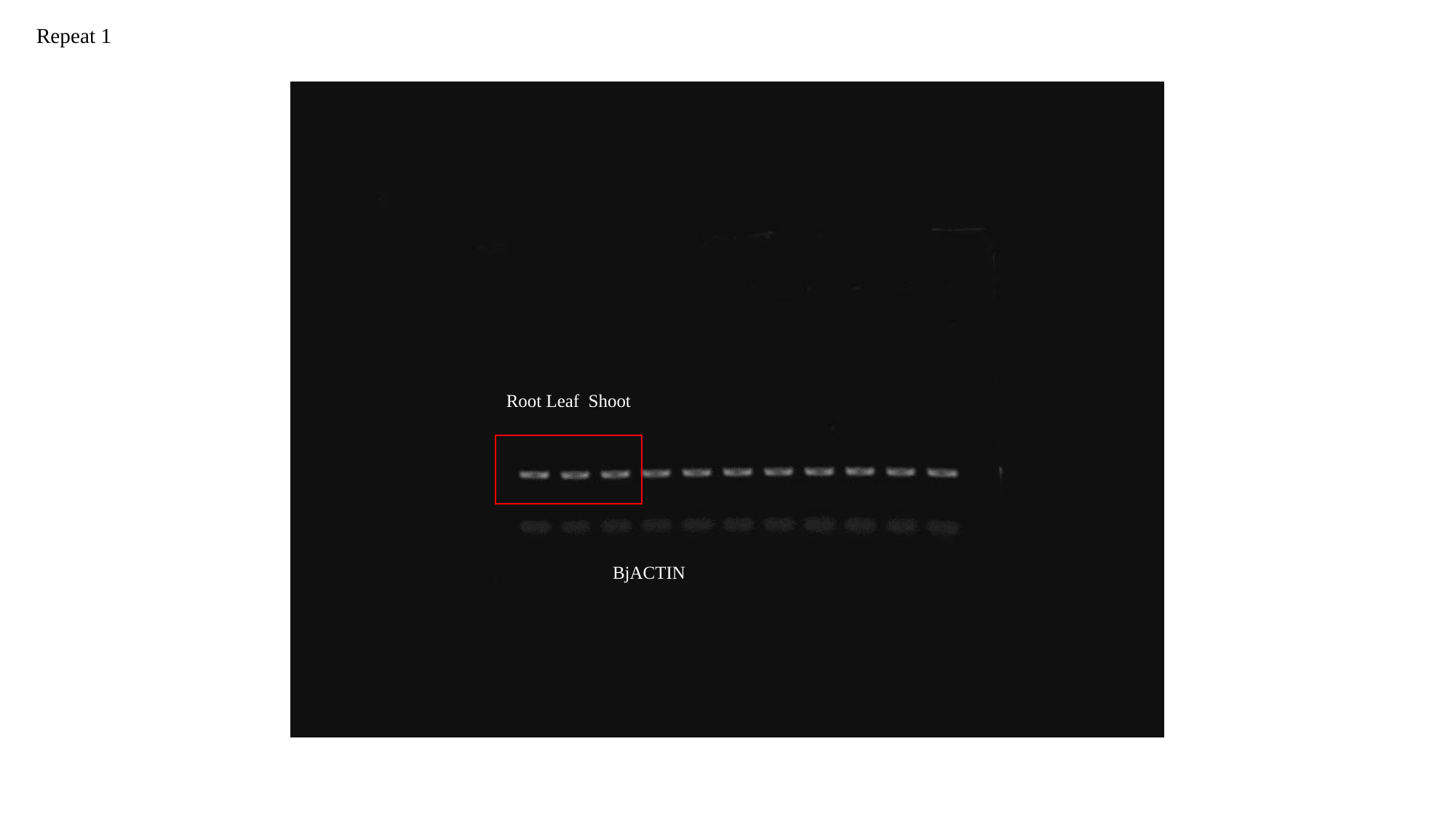

Repeat 1
Root Leaf Shoot
BjACTIN

## Slide 4
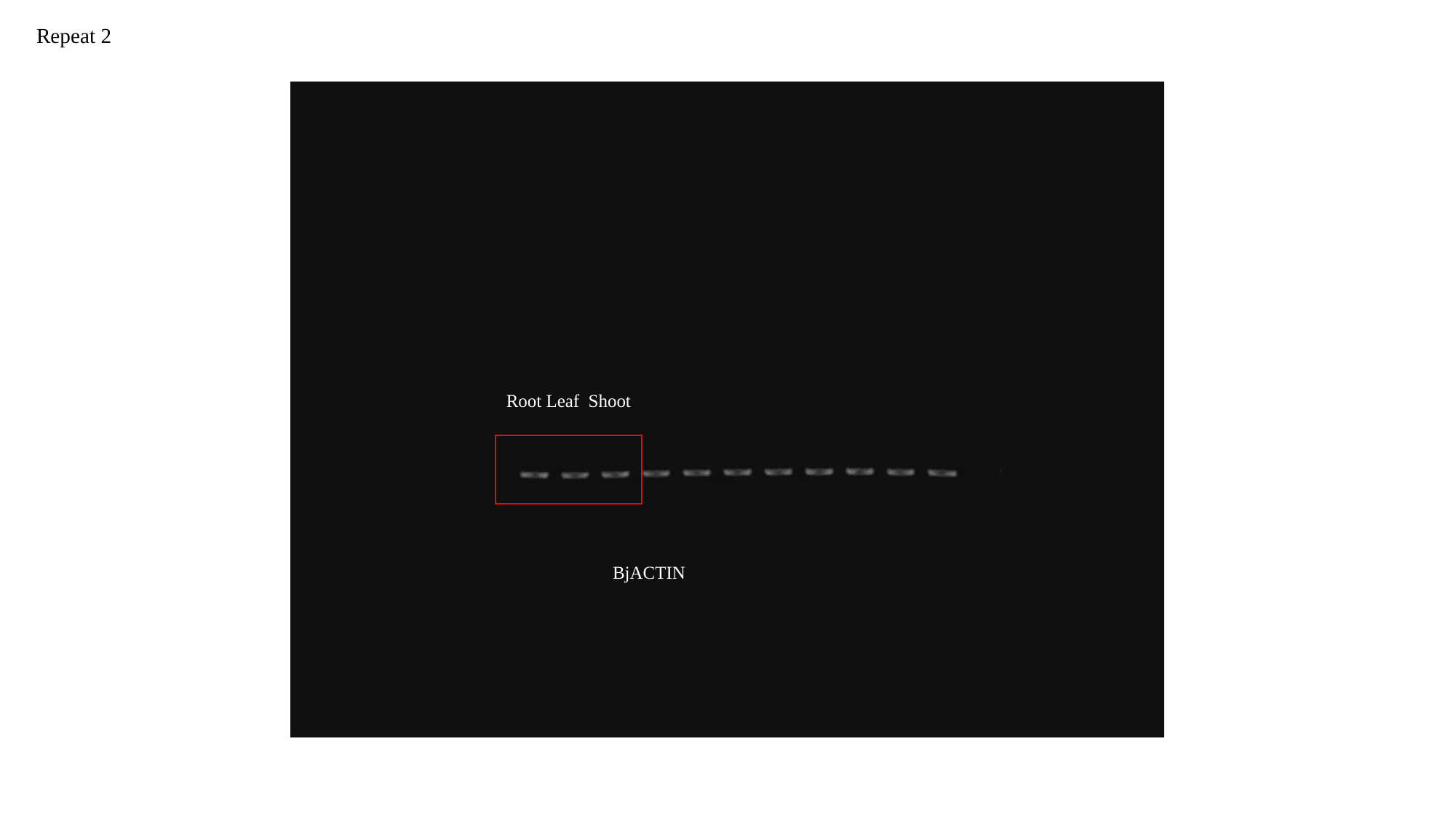

Repeat 2
Root Leaf Shoot
BjACTIN
